# The role of acetogens in microbially influenced corrosion of steel

**DOI:** 10.3389/fmicb.2014.00268

**Published:** 2014-06-03

**Authors:** Jaspreet Mand, Hyung Soo Park, Thomas R. Jack, Gerrit Voordouw

**Affiliations:** Petroleum Microbiology Research Group, Department of Biological Sciences, University of CalgaryCalgary, AB, Canada

**Keywords:** microbially influenced corrosion, acetogens, methanogens, sulfate-reducing bacteria, microbial community, pyrosequencing

## Abstract

Microbially influenced corrosion (MIC) of iron (Fe^0^) by sulfate-reducing bacteria (SRB) has been studied extensively. Through a mechanism, that is still poorly understood, electrons or hydrogen (H_2_) molecules are removed from the metal surface and used as electron donor for sulfate reduction. The resulting ferrous ions precipitate in part with the sulfide produced, forming characteristic black iron sulfide. Hydrogenotrophic methanogens can also contribute to MIC. Incubation of pipeline water samples, containing bicarbonate and some sulfate, in serum bottles with steel coupons and a headspace of 10% (vol/vol) CO_2_ and 90% N_2_, indicated formation of acetate and methane. Incubation of these samples in serum bottles, containing medium with coupons and bicarbonate but no sulfate, also indicated that formation of acetate preceded the formation of methane. Microbial community analyses of these enrichments indicated the presence of *Acetobacterium*, as well as of hydrogenotrophic and acetotrophic methanogens. The formation of acetate by homoacetogens, such as *Acetobacterium woodii* from H_2_ (or Fe^0^) and CO_2_, is potentially important, because acetate is a required carbon source for many SRB growing with H_2_ and sulfate. A consortium of the SRB *Desulfovibrio vulgaris* Hildenborough and *A. woodii* was able to grow in defined medium with H_2_, CO_2_, and sulfate, because *A. woodii* provides the acetate, needed by *D. vulgaris* under these conditions. Likewise, general corrosion rates of metal coupons incubated with *D. vulgaris* in the presence of acetate or in the presence of *A. woodii* were higher than in the absence of acetate or *A. woodii*, respectively. An extended MIC model capturing these results is presented.

## Introduction

Corrosion failures occur in water-transporting pipelines, when the anodic dissolution of iron (Fe^0^ → Fe^2+^ + 2e^−^) is coupled to the cathodic reduction of oxygen (2H^+^ + ½O_2_ + 2e^−^ → H_2_O). Pipelines are generally subject to high corrosion rates, if oxygen is readily available as an electron acceptor. To limit corrosion, water transported through these pipelines is degassed and is kept anaerobic through the addition of chemical oxygen scavengers such as sodium bisulfite (SBS). Protons are reduced at the cathode (2H^+^ + 2e^−^ → H_2_), instead of oxygen, under the resulting anaerobic conditions. This generally results in much lower corrosion rates, e.g., with linear polarization resistance (LPR) abiotic carbon steel corrosion rates of 0.26 and 0.11 mm/yr were observed under aerobic and anaerobic conditions, respectively, in a defined medium (Caffrey et al., 2008).

Hydrogenotrophic microorganisms consume cathodic hydrogen in a process that may contribute to microbially influenced corrosion (MIC). Sulfate-reducing bacteria (SRB), using cathodic hydrogen to reduce sulfate to sulfide, are often associated with MIC. A mechanism, in which corrosive SRB remove electrons directly from the iron surface, has been proposed recently (Dinh et al., 2004; Enning et al., 2012). The resulting sulfide precipitates as characteristic, black iron sulfide, whereas the excess ferrous iron forms precipitates as iron carbonate, when bicarbonate is present (4Fe^0^ + 5H^+^ + 3HCO^−^_3_ + SO^2−^_4_ → FeS + 3FeCO_3_ + 4H_2_O).

**Figure 1:**
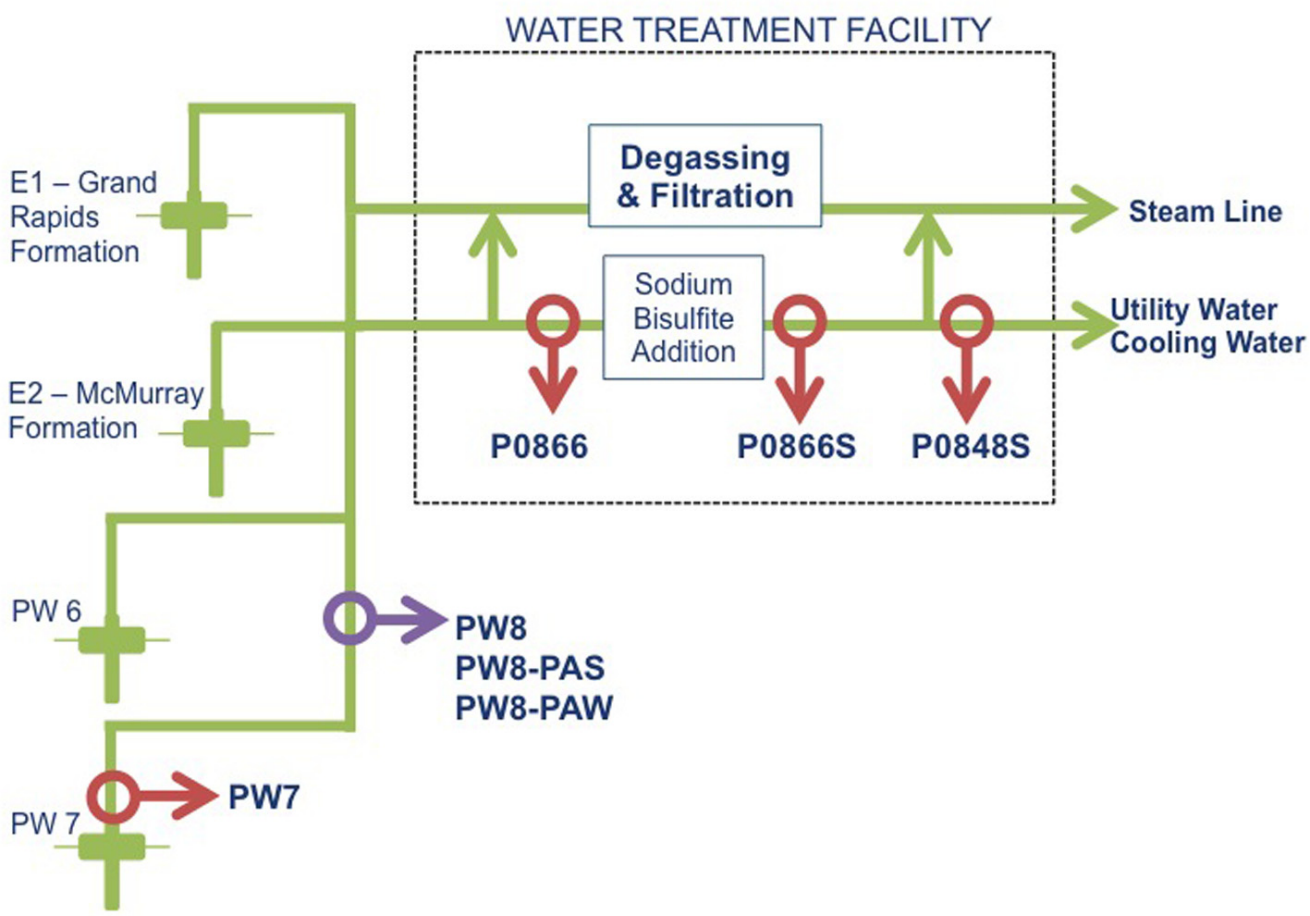
**Schematic diagram of the water pipeline system from the SAGD site**. Samples were collected at various points. Water was drawn up (PW7), taken to a water treatment facility (P0866) and treated with sodium bisulfite (P0866S and P0848S). A corrosion failure led to the collection of a pipe sample (PW8-PAS), which was transported with associated water (PW8-PAW). A water sample was also taken at this point (PW8).

In the absence of sulfate, hydrogenotrophic methanogens can contribute to MIC by catalyzing 4Fe^0^ + 5H^+^ + 5HCO^−^_3_ → CH_4_ + 4FeCO_3_ + 3H_2_O (Dinh et al., 2004; Uchiyama et al., 2010). Methanogenesis with associated MIC was also demonstrated in a pipeline system, which transports brackish water (~5 g/L of NaCl) from the Grand Rapids and McMurray (GM) formations (Figure 1) for use in bitumen production by steam assisted gravity drainage (SAGD), as shown by Park et al. (2011). This water lacked sulfate, but contained high concentrations of bicarbonate. SBS was injected in this system to decrease corrosion rates. Interestingly, injection of SBS led to a drastic change in microbial community composition, which was dominated by *Desulfocapsa* (class *Deltaproteobacteria*) downstream from the SBS injection point (Park et al., 2011). *Desulfocapsa* derives energy for growth from the disproportionation of bisulfite into sulfate and sulfide (Finster et al., 2013) and this organism clearly took advantage of a situation where low concentrations of bisulfite are provided continuously.

During further studies of MIC-associated methanogenesis at this SAGD water distribution system we found that concentrations of acetate of up to 1 mM were also produced, suggesting involvement of hydrogenotrophic acetogens, catalyzing (4Fe^0^ + 6HCO^−^_3_ + 5H^+^ → CH_3_COO^−^ + 4FeCO_3_ + 4H_2_O). These microorganisms normally produce acetate from hydrogen and carbon dioxide (Hu et al., 2013; Straub et al., 2014). We obtained another set of samples from a fresh water well from the same SAGD site (Figure 1: PW7). Water samples from this well had low concentrations of NaCl (0–200 ppm) and sulfate (0–64 ppm) and high concentrations of bicarbonate (500 ppm), as indicated in Table 1. These were used to further explore the possible production of acetate from steel as a novel MIC reaction.

**Table 1:** **Water chemistry of samples obtained for this study**.

**Sample**	**pH**	**Sulfate**	**Sulfide**	**Ferrous iron**	**Acetate**	**Bicarbonate**^a^	**Sodium**^a^
PW7	7.59	0	0.5	4.64	0	530	81
P0866	7.51	64.2	0.36	0	0	510	60
P0866S	7.32	5.08	0.03	5.3	0	520	100
P0848S	7.47	5.95	0.03	3.3	0	530	230
PW8	6.73	34.87	0	2.33	12.15	ND^b^	ND^b^
PW8-PAW	6.89	26.52	0	5.65	6.17	ND^b^	ND^b^
PW8-PAS	6.71	62.36	1.52	1546	18.66	ND^b^	ND^b^

*All concentrations are in ppm (mg/L)*.

a Data provided by company;

b *Not determined*.

## Materials and methods

### Field samples

Field samples were acquired from the utility water system at a SAGD site in northern Alberta. Water samples (2 L) were shipped in 1 L Nalgene (VWR International, Edmonton, AB) bottles filled to the brim to exclude oxygen. Sample PW7 was a freshwater sample from near the well-bore region with a temperature of 11°C. Sample P0866, was immediately upstream of the water treatment facility, where SBS was injected and was between 20 and 25°C. Samples P0866S and P0848S were taken downstream from this facility (Figure 1). All samples were stored in a Coy (Grass Lake, MI) anaerobic hood with an atmosphere of 90% N_2_ and 10% CO_2_ (N_2_-CO_2_, Praxair, Calgary, AB). A pipe segment representing a corrosion failure was also obtained, along with water sample PW8 from this site (Figure 1). The pipe segment was shipped in a bucket, together with pipe-associated water (PW8-PAW). Pipeline associated solids (PW8-PAS) were obtained by scraping the pipe segment in the anaerobic hood and suspending the scrapings in 0.2 μm filtered PW8-PAW, representing 10% of the internal volume of the pipe segment, as described elsewhere (Park et al., 2011). Upon arrival, 1 L of each water sample was filtered using a 0.2 μm Millipore nylon membrane (VWR International, Edmonton, AB) to collect biomass. This was immediately frozen at −80°C for use in DNA extraction.

While some water chemistry data were provided with the samples (bicarbonate, sodium), their chemical composition was characterized further. Sulfide concentrations were assessed colorimetrically using *N, N*-dimethyl-*p*-phenylenediamine (Trüper and Schlegel, 1964). Aqueous ferrous iron concentrations were assessed using a colorimetric assay using ferrozine (Park et al., 2011). Ammonium concentrations were assayed using the indophenol method and the pH of the samples was measured using a Thermo Scientific Inc. Orion 370 model pH meter (VWR International, Mississauga, ON). To measure acetate concentrations, a high-performance liquid chromatograph (HPLC) from Waters (Mississauga, ON), model 515, equipped with a Waters 2487 model UV detector set at 220 nm and an organic acid column (250 × 4.6 mm, Alltech Prevail, Guelph, ON) eluted with 25 mM KH_2_PO_4_ of pH of 2.5 was used. Samples (1 mL) were centrifuged at 13,300 rpm for 5 min, 300 μ L of the resulting supernatant was acidified using 20 μ L of 1 M H_3_PO_4_, and eluted at a flow rate of 0.8 mL/min. Sulfate concentrations were monitored using a Waters 600 model HPLC equipped with a Waters 432 conductivity detector and an IC-PAK anion column (150 × 4.6 mm, Waters) eluted with 24% (vol/vol) acetonitrile, 2% butanol, and 2% borate-gluconate concentrate. Following centrifugation 100 μ L of the sample supernatant was added to 400 μ L of the acetonitrile solution and eluted at 2 mL/min.

Two carbon steel coupons (American Society for Testing and Materials, ASTM a366, containing 0.015% carbon, 5 × 1 × 0.1 cm) were incubated with 50 mL of each water sample in a 120 mL serum bottle. The coupons were cleaned according to National Association of Corrosion Engineers (NACE) protocol RP0775-2005. The coupons were polished with 400 grit sandpaper, washed in a dibutylthiourea solution for 2 min, followed by a saturated bicarbonate solution for 2 min. The coupons were briefly rinsed in deionized water and then acetone and quickly dried in a stream of air. The coupons were weighed three times using an analytical balance and the average weight was recorded as the starting weight. The serum bottles were sealed with a headspace containing N_2_-CO_2_. During incubation, headspace methane and aqueous acetate concentrations were monitored. For methane measurements, 0.2 mL of the headspace was sampled periodically using a syringe that had been flushed with N_2_-CO_2_ and injected into a Hewlett-Packard (Mississauga, ON) model 5890 gas chromatograph at 150°C, equipped with a flame-ionizing detector set to 200°C and a stainless steel column (0.049 × 5.49 cm, Porapak R 80/100, Supelco, Oakville, ON). Following incubation, surface deposits were removed from each coupon using a non-scratching wipe. The coupons were cleaned according to the NACE protocol and again weighed three times. The weight loss (Δ W) was converted into a general corrosion rate (CR, mm/year) by the equation: *CR* = 87600 × Δ *W*/*A* × *T* × *D* where *A* is the coupon surface area (cm^2^), *T* is the incubation time (hours), and *D* is steel density (7.85 g/cm^3^), as described previously (Park et al., 2011).

### Field sample enrichments

Two carbon steel coupons (ASTM a366) were incubated in 50 mL of anaerobic Coleville synthetic brine (CSB-K) medium (Callbeck et al., 2013) in sealed 120 mL serum bottle microcosms with N_2_-CO_2_. Each microcosm was anaerobically inoculated with 2.5 mL of each field sample (5% inoculum of field sample). As a control, two corrosion coupons were incubated anaerobically with 50 mL of medium only. The incubation periods lasted between 8 and 16 weeks at 32°C, under constant shaking at 100 rpm. During this time, concentrations of methane were measured using gas chromatography and concentrations of acetate were measured using HPLC. For incubations with PW8, PW8-PAW, or PW8-PAS only corrosion rates were determined from metal weight loss. For incubations involving samples PW7, P0866, P0866S, and P0848S, biomass was also collected from each microcosm. The metal coupons were removed from the microcosm and washed with CSB-K medium to remove any unattached material. The coupons were gently scraped with a blade and the resulting biomass was considered representative of the biofilm population. The coupons were then cleaned according to the NACE protocol, and general corrosion rates were determined.

### DNA isolation from the field samples and enrichments

Biomass, collected from each field sample and from the biofilm fractions, was used for DNA extraction. DNA was extracted, using a bead-beating procedure outlined by the manufacturer of the FastDNA® Spin Kit for Soil (MP Biomedicals, Santa Ana, CA). DNA was eluted using 10 mM Tris-Cl pH 8.5 (Buffer EB from the QIAquick kit, Qiagen, Toronto, ON). The concentration of DNA extracted from each sample was quantified using the Qubit Fluorometer, and Quant-iT™ dsDNA HS Assay Kit (Invitrogen, Burlington, ON).

### Community analysis by pyrosequencing

PCR amplification of 16S rRNA genes from isolated community DNA was performed using primers 926Fw (AAACTYAAAKGAATTGRCGG) and 1392R (ACGGGCGGTGTGTRC) using the Taq Polymerase I Kit (Qiagen, Toronto, ON). PCR was for 5 min at 95°C, followed by 20 cycles of 30 s at 95°C, 45 s at 55°C, and 90 s at 72°C and finally 7 min at 72°C. A second PCR step used FLX titanium amplicon primers 454_RA_X and 454T_FwB for an additional 10 cycles. These have the sequences for primers 926Fw and 1392R at their 3′ ends. Primer 454T_RA_X has a 25 nucleotide A-adaptor (CGTATCGCCTCCCTCGCGCCATCAG) and a 10 nucleotide multiplex identifier barcode sequence X. Primer 454T_FwB has a 25 nucleotide B-adaptor sequence (CTATGCGCCTTGCCAGCCCGCTCAG). The resulting 16S rRNA PCR amplicons were purified and sent for pyrosequencing to the Genome Quebec and McGill University Innovation Centre (Montreal, QC). Pyrosequencing was done using a Genome Sequencer FLX Instrument, using a GS FLX Titanium Series Kit XLR70 (Roche Diagnostics Corporation, Laval, QC). Pyrosequencing and bioinformatic analysis of the sequences obtained have all been described elsewhere (Park et al., 2011; Soh et al., 2013).

### Analysis of pure cultures of an SRB and an acetogen

*Desulfovibrio vulgaris* Hildenborough and *Acetobacterium woodii* (DSM 1030) were used as model SRB and acetogen strains, respectively and were grown in Widdel-Pfennig (WP) medium with 10 mM sulfate and 30 mM NaHCO_3_ (Caffrey et al., 2007). Microcosms were inoculated with a culture of *A. woodii*, a culture of *D. vulgaris*, a co-culture of both strains, or no inoculum. A fifth microcosm containing a culture of *D. vulgaris* was amended with 3 mM acetate. Incubations were in 160 mL serum bottles containing 40 mL of medium and a headspace of 80% H_2_ and 20% CO_2_ Growth was measured by determining the optical density at 600 nm using a Shimadzu model 1800 UV spectrophotometer (Mandel, Guelph, ON) and by monitoring concentrations of acetate, sulfate, and sulfide.

To test corrosivities of these cultures, two carbon steel corrosion coupons (ASTM a366) were added to each of the microcosms. The headspace of these microcosms was either 80% H_2_ and 20% CO_2_, 16% H_2_ and 84% CO_2_ or N_2_-CO_2_, as indicated. Concentrations of acetate and sulfate were measured during incubation. Following incubation, the coupons were cleaned as per NACE protocol, metal weight loss was determined and this was converted into general corrosion rates.

## Results

### Methane and acetate production from steel coupons

The samples obtained from the water gathering system for a SAGD facility are indicated in Figure 1. Relative to samples obtained previously from the GM formations (Park et al., 2011), these had lower concentrations of NaCl (0–200 ppm, compared to 4000–6000 ppm for GM) and higher concentrations of sulfate (0–64 ppm, as compared to 0–2 ppm for GM). Both sets of samples had high bicarbonate concentrations (520 ppm, as compared to 400–1600 ppm for GM). Sulfide (0–1.5 ppm), ammonium (not shown) and acetate (0–19 ppm) were present in low concentrations (Table 1). Ferrous iron was low, except in the pipe-associated solids sample (Table 1: PW8-PAS), which had 1546 ppm, indicative of corrosion (Park et al., 2011).

**Figure 2:**
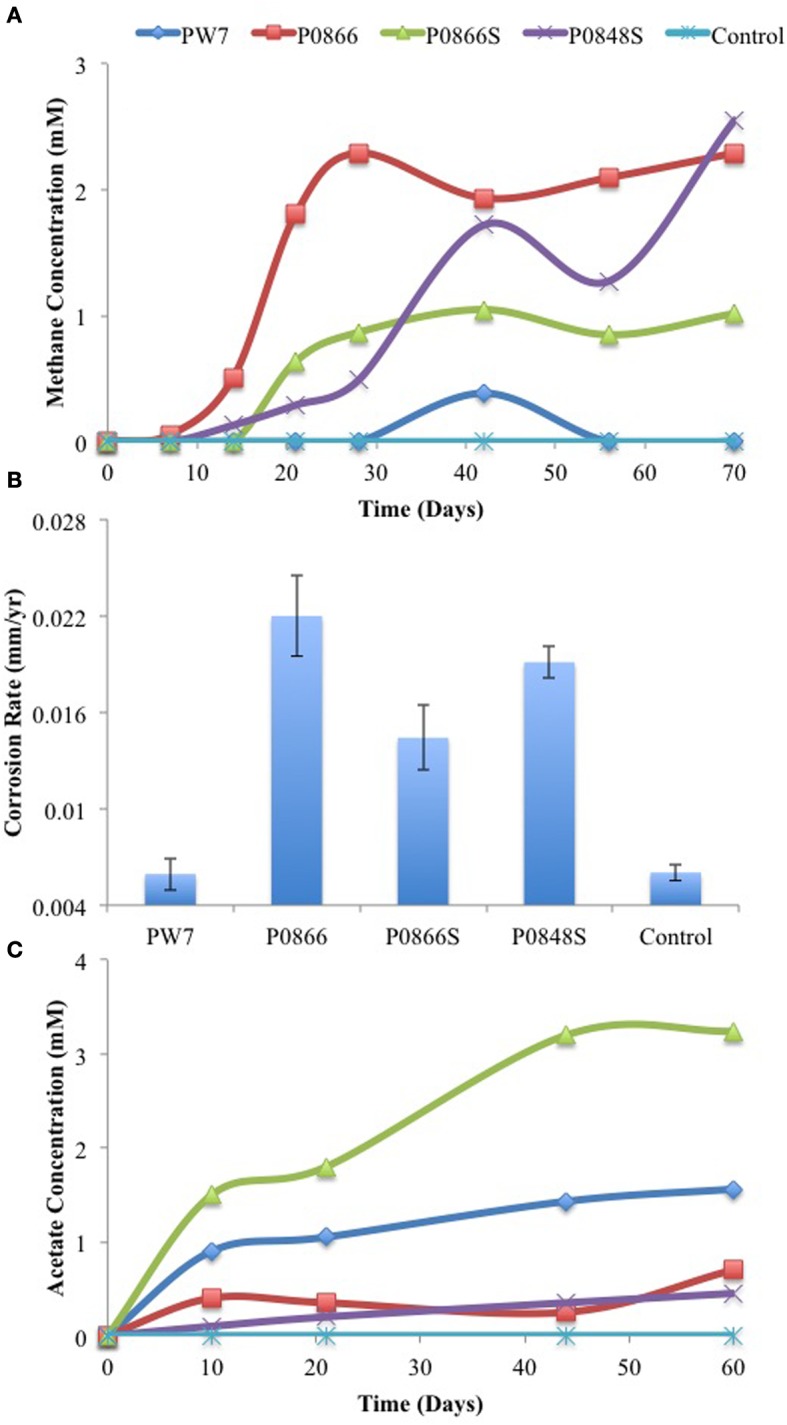
**Methane production (A) was monitored as field samples were incubated in the presence of carbon steel coupons and the associated corrosion rates (B) were determined using metal weight loss**. Acetate production **(C)** was monitored in an independent incubation of these field samples in the presence of carbon steel coupons. Data represent results from separate incubations with two coupons each.

Incubation of samples P0866, P0866S, and P0848S with carbon steel coupons and a headspace of N_2_-CO_2_ for 70 days gave formation of 2.3, 1.0, or 2.6 mM headspace methane, respectively (Figure 2A). No significant methane was formed in incubations with sample PW7 or the water-only control. Weight loss corrosion rates were higher (*p* < 0.00032) for incubations with samples P0866, P0866S, and P0848S (Figure 2B: average 0.018 ± 0.0040 mm/yr, *n* = 6) than with sample PW7 or the water-only control (Figure 2B: average 0.0060 ± 0.00091 mm/yr, *n* = 4). The formation of acetate was evaluated in a separate experiment indicating formation of 0.45 to 3.2 mM acetate in the aqueous phase of these incubations, whereas no acetate was formed in the water-only control (Figure 2C). Neither methane nor acetate were formed in the absence of steel coupons (results not shown).

**Figure 3:**
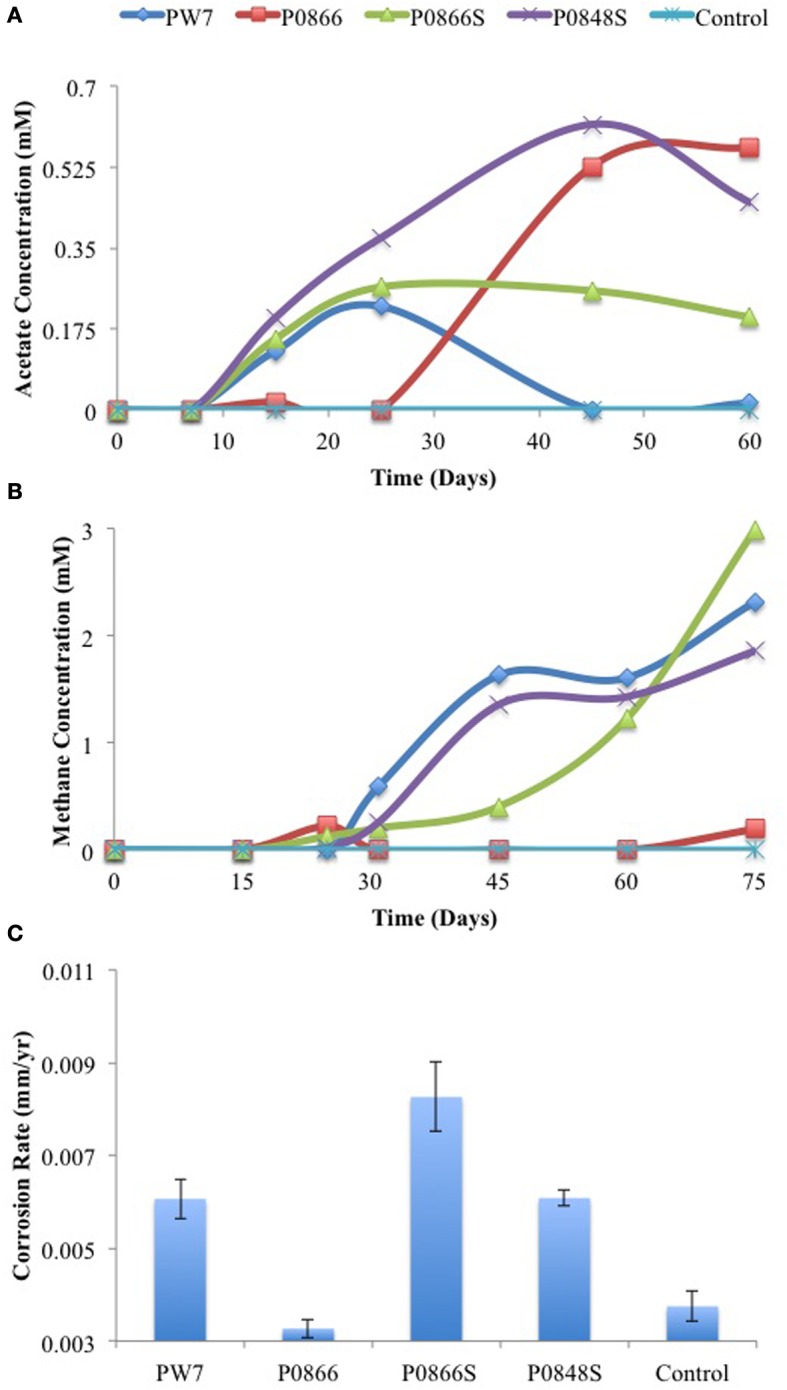
**Field samples were incubated in CSB-K medium with carbon steel coupons**. Acetate **(A)** and methane **(B)** concentrations were monitored during incubation. Corrosion rates **(C)** were determined from metal weight loss. Data represent results from separate incubations with two coupons each.

In a variation of this experiment serum bottles with 70 mL CSB-K medium, containing carbon steel coupons, 30 mM of bicarbonate and a headspace of N_2_-CO_2_ were inoculated with 3.5 ml of field sample. Acetate and methane were monitored as a function of time, and weight loss corrosion was determined at the end of the experiment. Production of up to 0.6 mM acetate was seen for all samples. This appeared to precede production of methane and go through a maximum (Figure 3A). Production of 1.9 to 3.0 mM headspace methane was observed with all samples, except P0866 (Figure 3B). The biofilms present on the coupons were isolated prior to processing of the coupons for measuring weight loss corrosion. The corrosion rates observed for incubations with samples PW7, P0866S, and P0848S (average 0.0068 ± 0.0012 mm/year, *n* = 6) were higher (*p* < 0.00076) than those with P0866 and the medium control (average 0.0035 ± 0.00031 mm/year, *n* = 4), as shown in Figure 3C. Hence, although both acetate and methane were formed as part of the corrosion process, the formation of headspace methane appeared to be correlated with the corrosion rate, i.e., P0866S, the sample with the highest corrosion rate (0.0083 mm/yr) also had the highest headspace methane concentration (3.0 mM).

**Figure 4:**
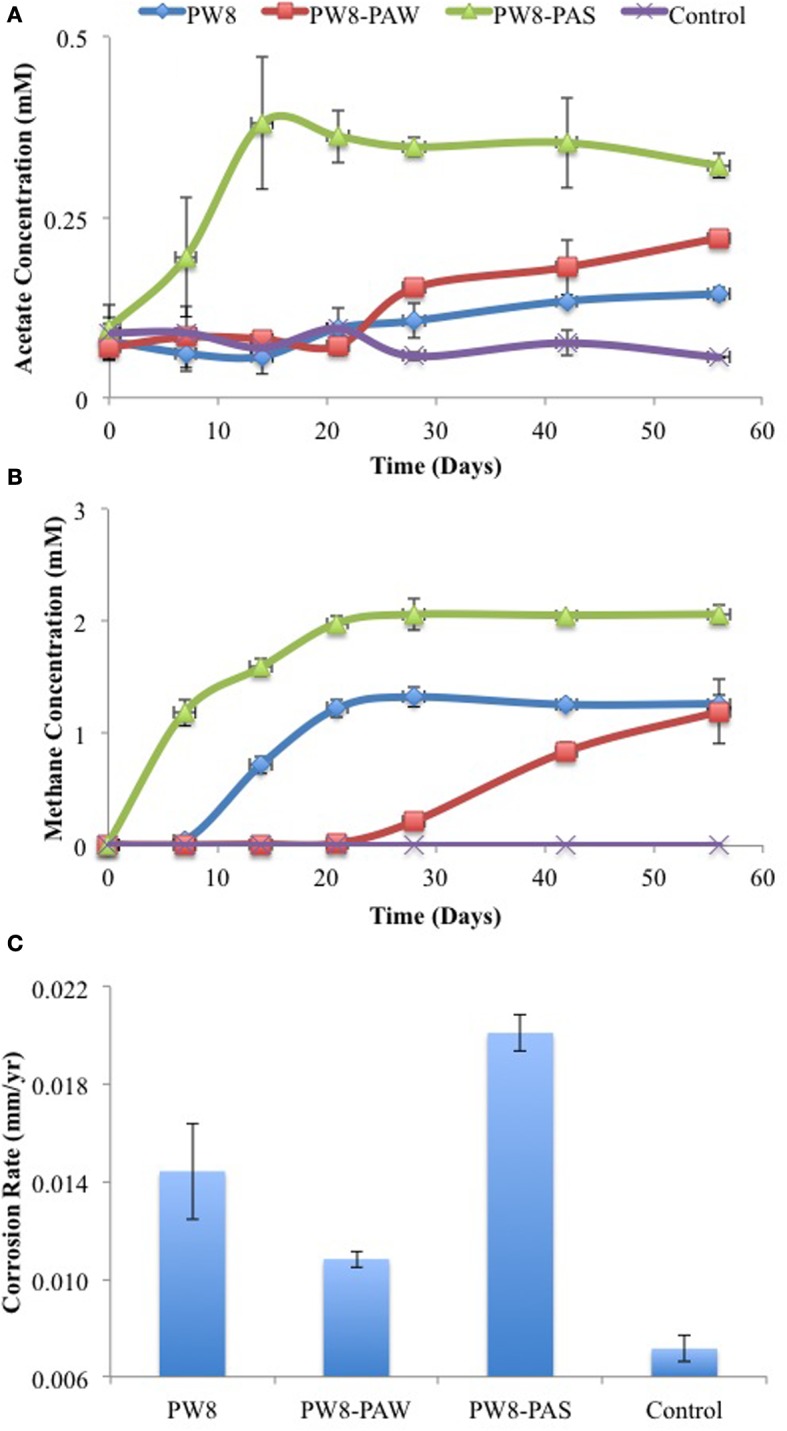
**Field samples (PW8, PW8-PAW, PW8-PAS) were incubated with carbon steel coupons**. Acetate **(A)** and methane **(B)** concentrations were monitored during incubation. Corrosion rates **(C)** were determined from metal weight loss. Data represent average results from two separate incubations with two coupons each.

**Figure 5:**
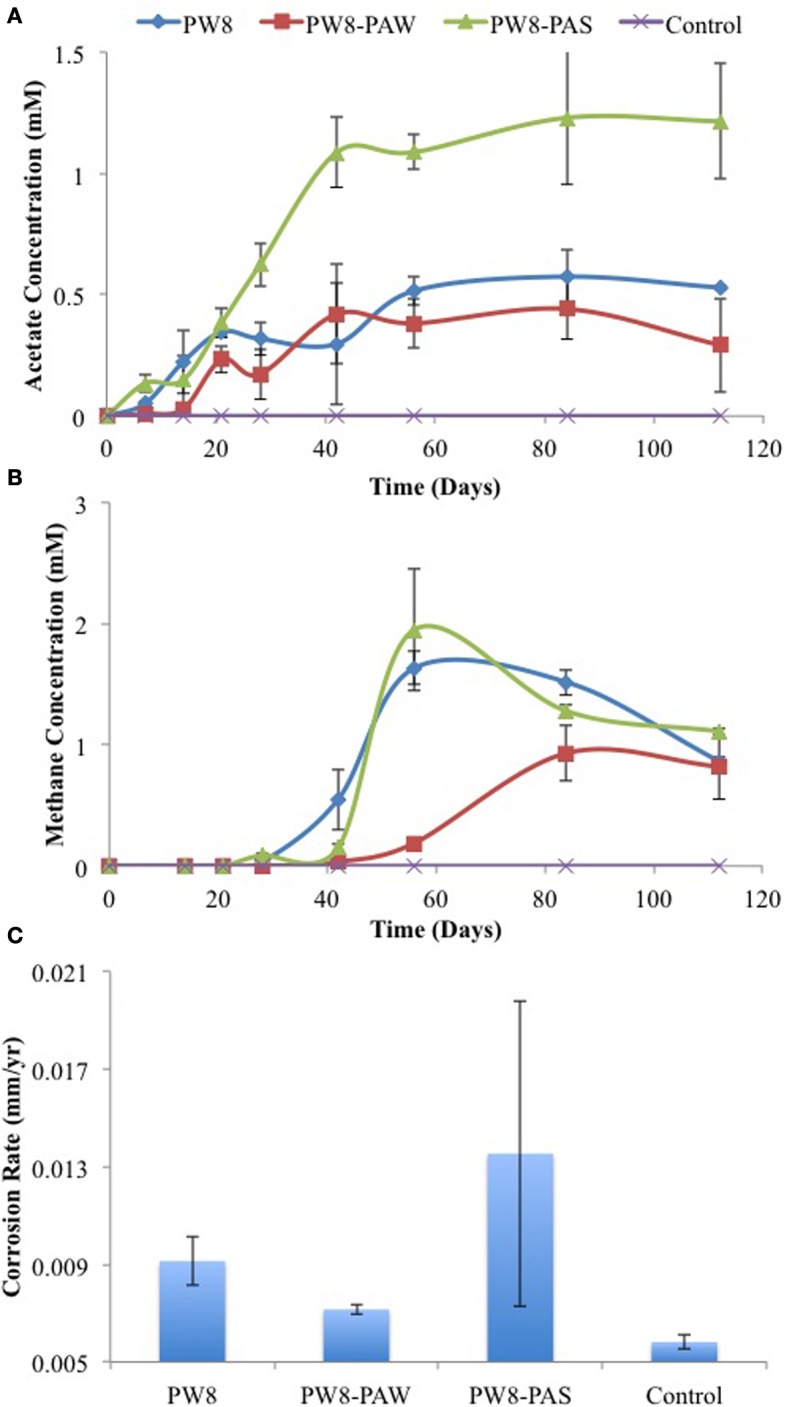
**Field samples were incubated in CSB-K medium with carbon steel coupons**. Acetate **(A)** and methane **(B)** concentrations were monitored during incubation. Corrosion rates **(C)** were determined from metal weight loss. Data represent average results from two separate incubations with two coupons each.

Similar results were obtained with samples PW8, PW8-PAW, and PW8-PAS, the latter representing pipe-associated solids suspended in microfiltered pipe-associated water (PW8-PAW). Incubation of metal coupons in these samples gave up to 0.38 mM acetate in the aqueous phase and 1 to 2 mM headspace methane (Figures 4A,B). The highest concentrations of acetate and methane were produced in incubations with PW8-PAS, which also had the highest corrosion rate (*p* < 0.0026) at 0.020 mm/yr (Figure 4C). When these field samples were inoculated in CSB-K medium, production of up to 1.2 mM acetate was seen to precede production of up to 2 mM of headspace methane (Figures 5A,B). Acetate and methane production were observed in incubations with PW8, PW8-PAW, and PW8-PAS, which had higher corrosion rates (*p* < 0.12) (average 0.0099 ± 0.0049 mm/yr, *n* = 12) than the uninoculated control (0.0058 ± 0.00042 mm/yr, *n* = 4) (Figure 5C).

### Microbial community compositions

**Figure 6:**
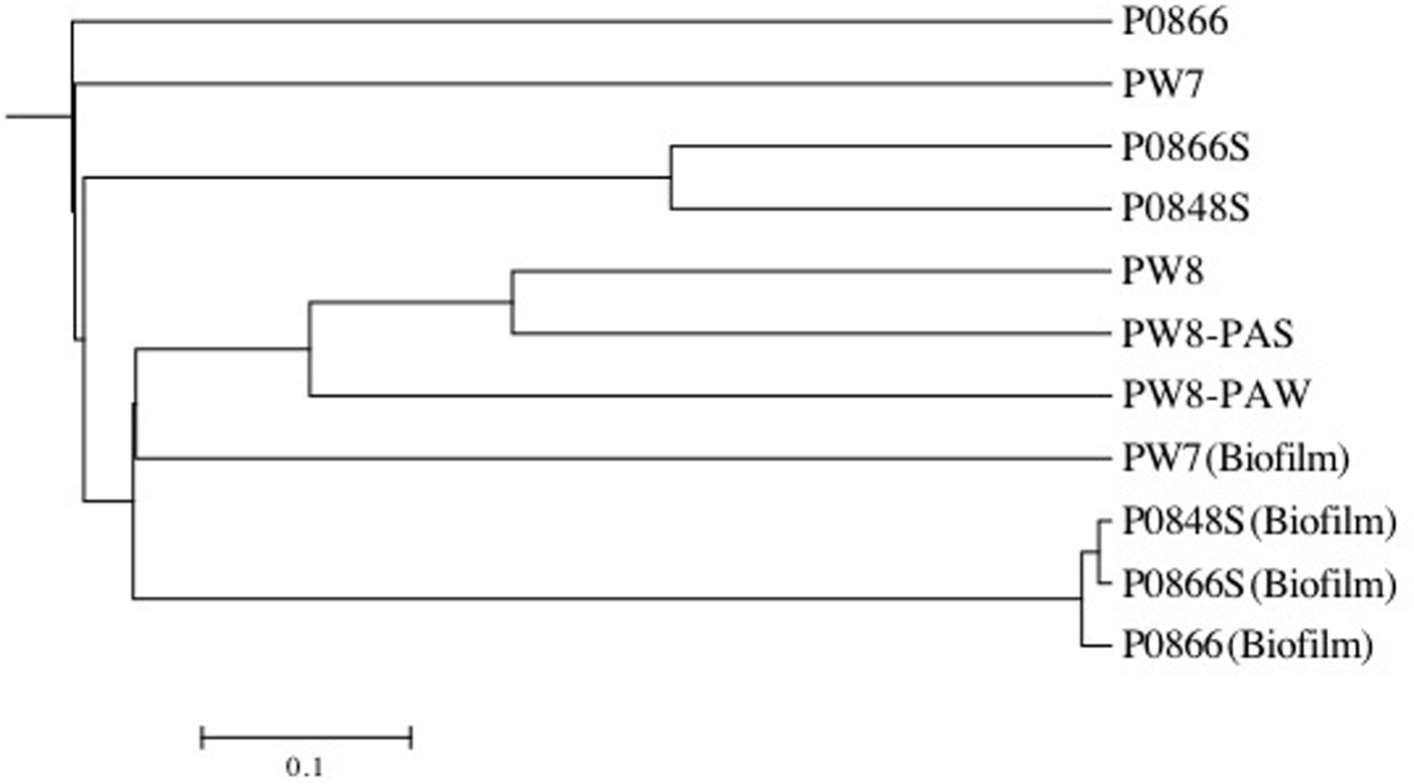
**Tree generated using UPGMA algorithm with distance between communities calculated using thetaYC coefficient in the Mothur software package**. Tree was visualized with Mega. All field samples formed a tree separate from all enrichment samples. Water field samples were distinct from pipeline-associated samples.

Pyrosequencing of 16S rRNA amplicons for all seven field samples and for four coupon enrichment biofilms, gave 1357 to 6417 quality controlled reads for each sample (Table 2). The compositions of the microbial communities derived from these results are compared in Figure 6. Although the water samples were all from a single pipeline system, it is evident that the microbial communities in water samples retrieved along the flow path were distinct. The community of PW7 near the fresh water producing well-differed from those of PW8 and P0866, both further downstream and all of these were distinct from the communities at P0866S and P0848S downstream from the SBS injection point (Figures 1, 6). Microbial communities of PW8-PAS and PW8-PAW formed a separate branch in the dendrogram together with that of PW8. Communities in coupon enrichment biofilms differed significantly from those in field samples (Figure 6). Microbial communities formed following incubation with P0866, P0866S, and P0848S were very similar, whereas PW7 (Biofilm) was distinct from this cluster (Figure 6).

**Table 2:** **Distribution of taxa (%) over high-quality reads**.

	**Fraction (%) of taxa for each sample**											**Fraction (%) of all reads**
	**PW7 V12_433**	**P0866 P0866**	**P0866S V12_460**	**P0848S V20_797**	**PW8 V22_924**	**PW8_PAW V22_925**	**PW8_PAS V22_926**	**PW7 (Biofilm) V15_602**	**P0866 (Biofilm) V15_607**	**P0866S (Biofilm) V15_608**	**P0848S (Biofilm) V15_609**	
**Number of high quality reads**	**4016**	**7093**	**6417**	**1357**	**4343**	**4403**	**4520**	**2427**	**2205**	**5474**	**3877**	
**TAXON**												
Family *Methanobacteriaceae*	0.00	**1.41**	0.00	0.59	**6.86**	0.18	**8.72**	0.21	**71.47**	**87.07**	**79.80**	**23.30**
Genus *Desulfocapsa*	**2.62**	0.41	**95.76**	**95.28**	**7.07**	**1.23**	**14.74**	0.82	0.27	0.02	0.05	**19.84**
Genus *Methanosaeta*	0.97	0.17	0.00	0.00	**14.46**	**16.53**	**18.30**	**17.14**	**7.08**	0.06	**2.71**	**7.04**
Genus *Pseudomonas*	0.62	0.03	0.05	0.00	0.09	0.20	0.07	**52.82**	**9.57**	**3.16**	**8.92**	**6.87**
Genus *Acidovorax*	**61.70**	0.01	0.20	0.00	**2.49**	0.61	0.04	0.04	0.14	0.04	0.08	**5.94**
Family *Rhodocyclaceae*	0.37	**48.23**	0.02	0.44	0.62	0.34	0.53	0.04	0.00	0.00	0.00	**4.60**
Genus *Dietzia*	0.00	0.00	0.00	0.00	0.00	**25.91**	0.09	0.00	0.00	0.00	0.00	**2.36**
Genus *Desulfurivibrio*	0.08	**23.52**	0.03	0.00	0.00	0.00	0.04	0.00	0.00	0.00	0.00	**2.15**
Genus *Acetobacterium*	0.08	0.01	0.00	0.00	0.55	0.25	**5.58**	**2.93**	0.05	**4.86**	**4.02**	**1.67**
Genus *Desulfovibrio*	0.92	**4.41**	0.03	**1.11**	0.12	0.09	**9.69**	0.25	0.23	0.02	0.08	**1.54**
Genus *Methanothermobacter*	0.00	0.11	0.00	0.07	**8.73**	**6.93**	0.11	0.00	0.00	0.00	0.00	**1.45**
Genus *Castellaniella*	0.00	0.00	0.00	0.00	**8.13**	**4.09**	**2.35**	0.00	0.00	0.00	0.00	**1.32**
Genus *Methanoculleus*	0.05	0.00	0.02	0.00	**3.36**	**4.77**	**3.94**	0.95	0.64	0.24	0.13	**1.28**
Genus *Desulfosporosinus*	0.15	0.00	0.00	0.00	0.18	0.11	**13.45**	0.00	0.00	0.00	0.00	**1.26**
Genus *Desulfobulbus*	0.87	0.55	0.13	0.00	**4.81**	**1.20**	**2.68**	0.41	0.14	0.00	0.03	0.98
Genus *Methanobacterium*	0.03	0.10	0.00	0.00	0.85	**1.23**	**1.17**	**4.29**	0.27	**2.25**	0.05	0.93
Genus *Geobacter*	**8.47**	0.00	0.00	0.00	0.05	0.02	0.00	0.00	0.00	0.02	0.00	0.78
Genus *Methanocalculus*	**1.12**	0.06	0.09	0.00	**1.75**	**1.98**	**2.08**	0.87	0.27	0.02	0.21	0.77
Genus *Marinobacterium*	0.30	0.00	0.02	0.00	0.60	0.89	**1.02**	**2.56**	**1.59**	0.00	0.59	0.69
Genus *Desulfomicrobium*	0.10	**5.43**	0.08	0.00	0.14	0.07	**1.02**	0.12	0.09	0.00	0.00	0.64
Phylum *Firmicutes*	0.25	**2.33**	0.02	0.00	0.65	0.80	0.55	0.21	0.18	0.00	0.03	0.45
Family *Rhodocyclaceae*	0.05	**1.04**	0.03	0.07	0.02	0.09	**3.23**	0.04	0.09	0.06	0.05	0.43
Genus *Methanolinea*	0.10	0.03	0.00	0.07	0.94	**1.75**	**1.13**	0.25	0.14	0.06	0.08	0.41
Genus *Comamonadaceae*	**3.36**	0.06	0.03	0.15	0.23	0.02	0.16	0.25	0.05	0.00	0.00	0.39
Genus *Marinobacter*	0.32	0.00	0.03	0.00	0.14	0.09	0.07	**1.85**	**1.27**	0.00	0.52	0.39
Phylum *Crenarchaeota*	0.27	0.00	0.00	0.07	2.37	**1.48**	0.00	0.00	0.00	0.00	0.00	0.38
Genus *Citricoccus*	0.00	0.00	0.00	0.00	0.00	**3.93**	0.00	0.00	0.00	0.00	0.00	0.36
Genus *Nitrospira*	0.00	0.00	0.00	0.00	**3.20**	0.50	0.00	0.00	0.00	0.00	0.00	0.34
Kingdom *Bacteria*	0.90	0.69	0.13	0.00	0.71	0.50	0.22	0.21	0.09	0.00	0.00	0.31
Genus *Gallionella*	**1.52**	0.00	0.05	0.00	**1.50**	0.18	0.00	0.00	0.00	0.00	0.00	0.30
Genus *Planococcus*	0.00	0.00	0.00	0.00	0.14	**3.04**	0.00	0.00	0.00	0.00	0.00	0.29
Genus *Methanolobus*	0.20	0.00	0.00	0.00	0.67	0.64	1.00	0.37	0.05	0.00	0.00	0.27
Sum	85.41	88.59	96.70	97.86	71.43	79.65	91.95	86.61	93.65	97.84	97.34	89.73

*Taxa in abundance greater than 1% are bolded. Taxa were ranked according to their overall abundance in the 16S rRNA amplicons for the samples. Data are for field samples (water) and the communities growing on steel coupons (biofilm) in incubations of field samples in medium with bicarbonate, but without sulfate. Only taxa with an average abundance in excess of 0.25% are shown*.

The microbial community of water sample PW7 was dominated by *Betaproteobacteria* of the genus *Acidovorax* (Table 2: 61.70%). This taxon had decreased to 2.49% of the community in water sample PW8, which also harbored significant fractions of methanogens of the class *Methanobacteriaceae* (16.44%), the genera *Methanosaeta* (14.46%) and *Methanoculleus* (3.36%), as well as of *Deltaproteobacteria* of the genera *Desulfocapsa* (7.07%) and *Desulfobulbus* (4.81%). The pipeline solids at this location, PW8-PAS, also harbored high fractions of *Deltaproteobacteria/Desulfovibrio* (9.69%), of *Firmicutes/Acetobacterium* (5.58%), and of *Firmicutes/Desulfosporosinus* (13.45%). Many of these taxa use hydrogen (and possibly Fe^0^) as electron donor for the reduction of CO_2_ to methane or to acetate (*Acetobacterium*) or for the reduction of sulfate to sulfide (*Desulfobulbus, Desulfosporosinus, Desulfovibrio*). The water associated with the pipe sample (PW8_PAW) had a high fraction of *Dietzia* (25.91%), which has been associated with hydrocarbon degradation (Bihari et al., 2011). The community composition of water sample P0866, taken further downstream, had high fractions of *Betaproteobacteria/Rhodocyclaceae* (48.23%), capable of nitrate reduction and of *Deltaproteobacteria* of the genera *Desulfurivibrio* (23.52%), *Desulfovibrio* (4.41%), and *Desulfomicrobium* (5.43%). Treatment with SBS gave rise to communities dominated by *Desulfocapsa* in water samples P0866S (95.76%) and P0848S (95.28%), which grows by disproportionation of bisulfite to sulfate and sulfide, as discussed elsewhere (Park et al., 2011). Hence, a succession of communities is present along the water flow path, with a very strong shift being observed by the addition of SBS. It is interesting that the microbial communities in the water samples, obtained from the water-transporting pipeline studied here, appeared to change so rapidly as a function of distance (Figure 6). This would seem to be at odds with the fact that the transport time of water from PW7 to P0848S is less than 1 day. These large changes in community composition may indicate that most biomass grows on the pipe walls as biofilms, is sloughed off at a high rate and appears to dominate the microbial community compositions seen. This explains why *Desulfocapsa* becomes a major community component immediately downstream from the SBS injection point (Table 2, Figure 1). The planktonic population would not be able to adjust in such a short time, but if the *Desulfocapsa* is biofilm-grown, it may continuously be swept into the flowing water.

Incubation of field samples with CSB-K medium, which contains CO_2_ and bicarbonate but lacks sulfate, in the presence of metal coupons led to their colonization by *Methanobacteriaceae*, which were present at 71.75, 89.31, and 79.86% in incubations with P0866, P0866S, and P0848. In the incubation PW7, only 4.49% of the reads were of the class *Methanobacteriaceae*. The latter biofilm had a high fraction of the acetotrophic methanogen *Methanosaeta* (17.14%), as well as of *Pseudomonas* (52.82%). Compared to the field samples, the biofilm communities also showed an increased presence of the acetogen *Acetobacterium* (Table 2). The colonization of carbon steel coupons by methanogens and acetogens (Table 2) can explain the production of acetate and methane observed in these incubations (Figure 3).

### Growth of and corrosion by a pure culture consortium of an SRB and an acetogen

**Figure 7:**
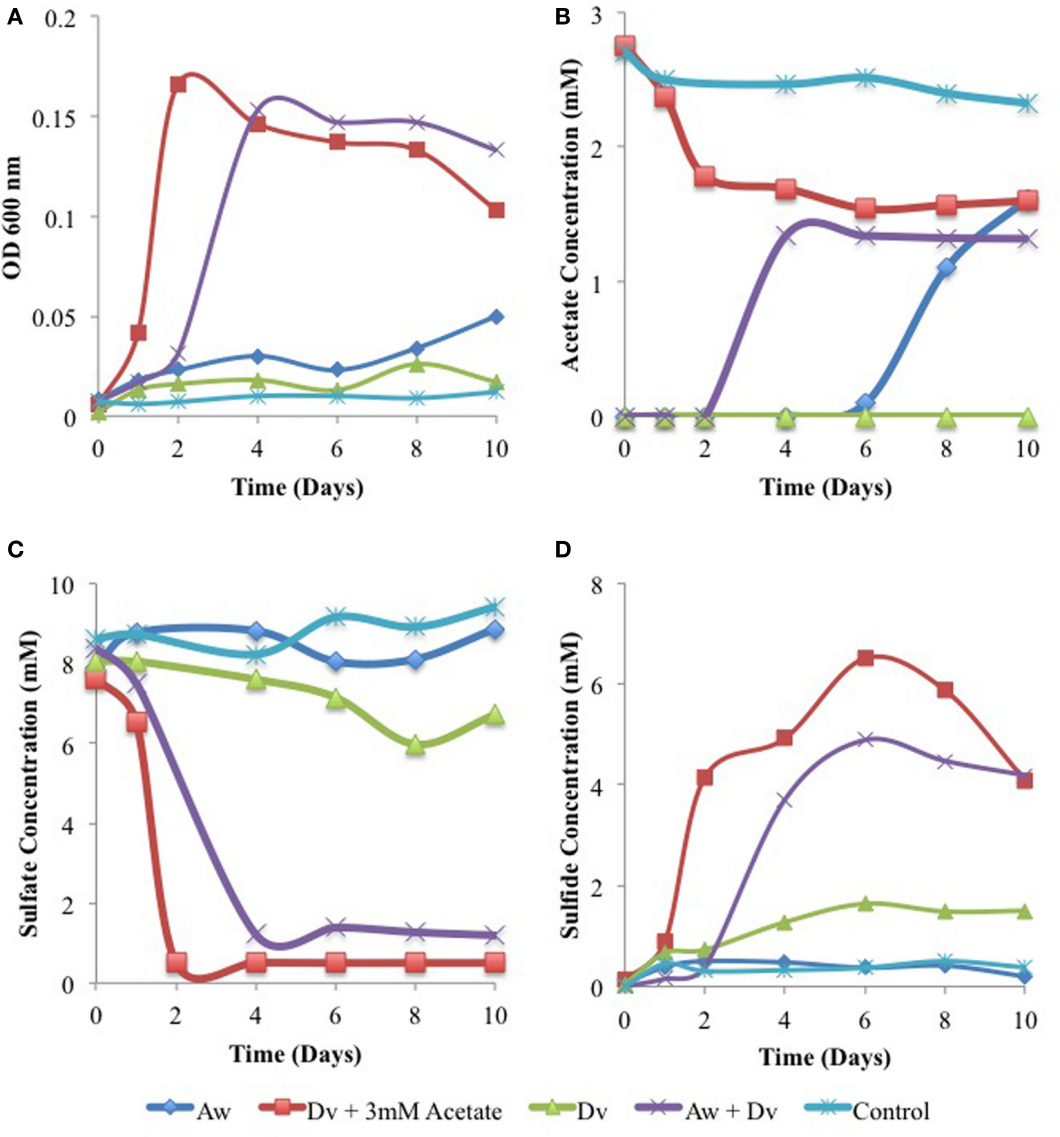
**Growth physiology of *D. vulgaris* (Dv) and *A. woodii* (Aw) monocultures and a co-culture in closed serum bottles under an atmosphere of 80% H_2_, 20% CO_2_**. Cell density **(A)**, acetate concentrations **(B)**, sulfate concentrations **(C),** and sulfide concentrations **(D)**. Data represent results from separate incubations without coupons present.

SRB of the genus *Desulfovibrio* or *Desulfomicrobium* need acetate as a carbon source when deriving energy for growth from reduction of sulfate with H_2_ or Fe^0^ (Badziong et al., 1979; Dinh et al., 2004; Caffrey et al., 2007; Enning et al., 2012). When acetate is absent or limiting, this can in principle be provided by acetogens from H_2_ (or Fe^0^) and CO_2_. In order to test this hypothesis we studied the growth of the model SRB *Desulfovibrio vulgaris* Hildenborough and the model acetogen *Acetobacterium woodii* in WP medium, containing bicarbonate and sulfate and a headspace of 80% H_2_ and 20% CO_2_. A monoculture of *A. woodii* grew poorly in this medium as judged by turbidity (Figure 7A) and produced up to 1.6 mM acetate after 10 days (Figure 7B). A monoculture of *D. vulgaris* did not grow (Figure 7A), did not have detectable acetate (Figure 7B) and reduced only 2 mM sulfate to sulfide (Figures 7C,D). Addition of 3 mM acetate to the monoculture of *D. vulgaris* gave strong growth, sulfate reduction, and sulfide production, while using 1.5 mM of added acetate. Following a lag phase of 2 days needed for the production of 1.3 mM acetate (Figure 7B), the co-culture of *D. vulgaris* and *A. woodii* exhibited similar growth, sulfate reduction and sulfide production as the monoculture of *D. vulgaris* with added acetate. No sulfate reduction or acetate production was seen in the inoculum-free control. Hence, *A. woodii* can provide the acetate needed by *D. vulgaris* for growth under chemolithotrophic conditions.

**Figure 8:**
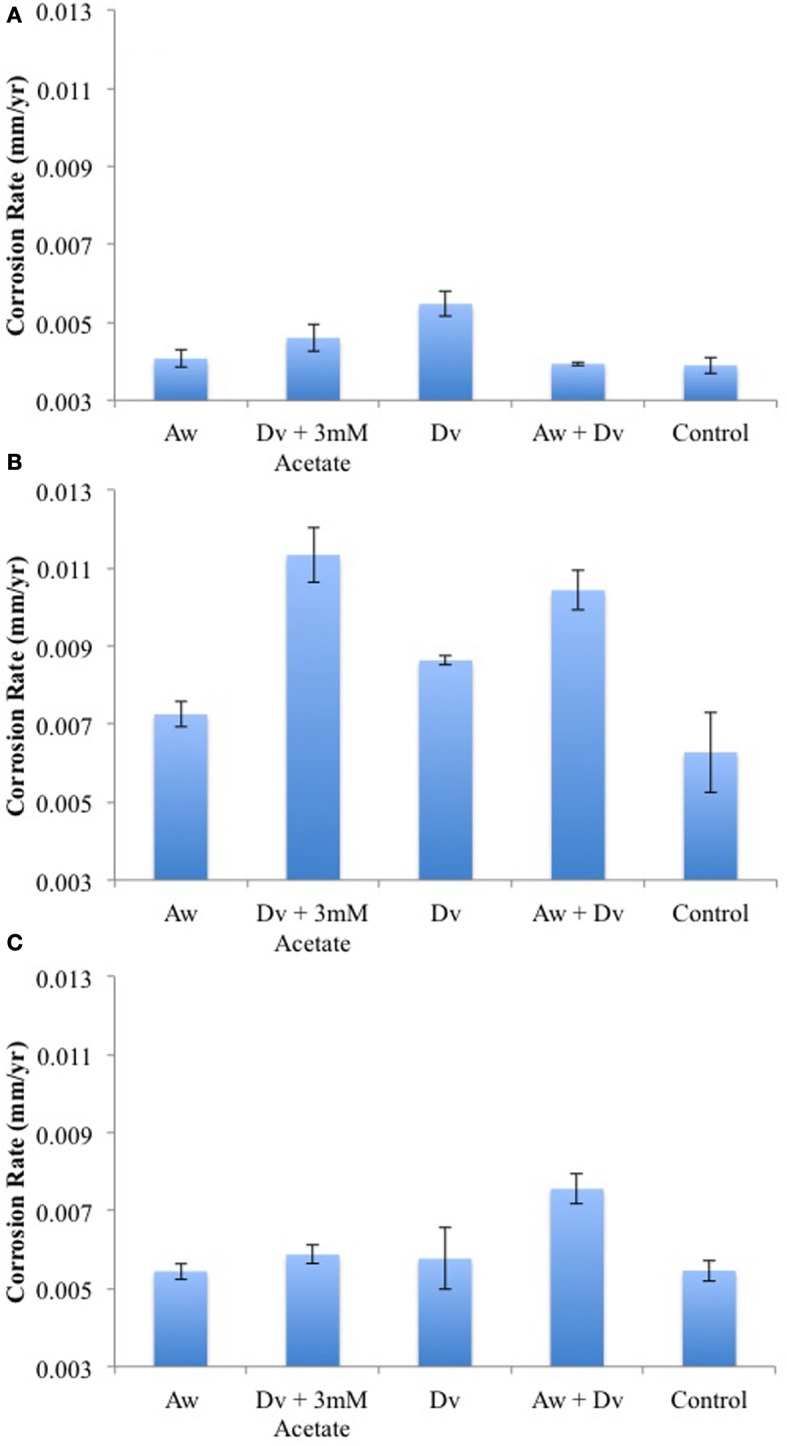
**Corrosion rates of carbon steel coupons incubated in closed serum bottles with monocultures and a coculture of *A. woodii* (Aw) and *D. vulgaris* (Dv) an atmosphere of either (A) 90% N_2_, 10% (B) 16% H_2_, 84% CO_2_,or (C) 80% H_2_, 20% CO_2_**. Control bottles had no inoculum. Data represent average results from two separate incubations with two coupons each.

When carbon steel coupons were added to serum bottles, containing WP medium with sulfate and bicarbonate and a headspace of N_2_-CO_2_ and inoculated with *D. vulgaris*, *D. vulgaris* and 3 mM acetate, *A. woodii*, *D. vulgaris*, and *A. woodii*, or no inoculum, similar corrosion rates were observed of 0.0039 to 0.0055 mm/yr in all incubations (Figure 8C). This indicates that Fe^0^ alone is not a good electron donor for sulfate or carbon dioxide reduction by these two type cultures. In order to determine whether Fe^0^ can be used as an electron donor co-metabolically with H_2_, the experiment was repeated with a headspace of 80% (vol/vol) H_2_ and 20% CO_2_ (excess H_2_ for reduction of 10 mM sulfate) or 16% H_2_ and 84% CO_2_ (insufficient H_2_ for reduction of 10 mM sulfate). The highest corrosion rates were observed with limiting H_2_ for incubations with *D. vulgaris* and 3 mM acetate (0.0113 mm/yr) or with the coculture of *D. vulgaris* and *A. woodii* (0.0104 mm/yr), as shown in Figure 8B. These were higher (*p* < 0.000068) than the corrosion rates observed for incubations with *D. vulgaris* alone without added acetate, with *A. woodii* alone or with the uninoculated control (average 0.0074 ± 0.0015 mm/yr, *n* = 12). In the presence of excess H_2_ lower corrosion rates, between 0.0054 and 0.0059 mm/yr, were observed for all incubations except for the co-culture, which was somewhat higher (0.0076 mm/yr), as shown in Figure 8A. Overall, these results indicate that the co-culture of *D. vulgaris* and *A. woodii* can co-metabolically corrode carbon steel at a rate comparable to that when acetate is provided directly to the *D. vulgaris* culture.

## Discussion

The role of acetic acid and CO_2_ in the anaerobic corrosion of steel has been studied extensively. The presence of acetic acid is generally considered corrosive even at moderate concentrations (100 ppm). Although this may be credited to a pH-effect, since protons are consumed in cathodic corrosion reactions, it appears that acetate, the predominant form at higher pH, is also corrosive (Hedges and McVeigh, 1999; George et al., 2004; Liu et al., 2008). The potential role of microorganisms in the acetate-CO_2_ corrosion landscape has been reviewed by Suflita et al. (2008). Anaerobic consortia produce acetic acid and CO_2_ by fermentation of carbohydrates or of oil hydrocarbons (Suflita et al., 2008; Callbeck et al., 2013). Acetogenic bacteria, such as *A. woodii*, can also produce acetic acid from H_2_ and CO_2_, using the Wood-Ljungdahl pathway to synthesize acetyl-CoA (Schiel-Bengelsdorf and Dürre, 2012; Leang et al., 2013).

As shown here, anaerobic consortia in samples from a SAGD water gathering system were able to produce methane, but also acetate in the presence of steel coupons (Figures 2–5). The production of methane using iron as an electron donor for reduction of CO_2_ is well-known. Highly corrosive methanogens, which like highly corrosive SRB may be able to extract electrons directly from the steel surface, have been isolated (Daniels et al., 1987; Dinh et al., 2004; Uchiyama et al., 2010). However, the production of acetate from iron has not previously been demonstrated, although the possibility has been discussed (Phelps, 1991; Suflita et al., 2008). Acetate production was shown when coupons were incubated directly with field samples (Figures 2, 4) and when coupons were incubated with CSB-K medium inoculated with the same field samples. The concentration of acetate formed in the aqueous phase tended to be less than that of methane in the headspace. Acetate formation appeared to precede methane formation (Figure 3). The presence of significant fractions of the acetotrophic methanogen *Methanosaeta* in the coupon-attached biofilms (Table 2) suggests that the acetate formed by acetogens may subsequently be converted into methane and CO_2_.

**Figure 9:**
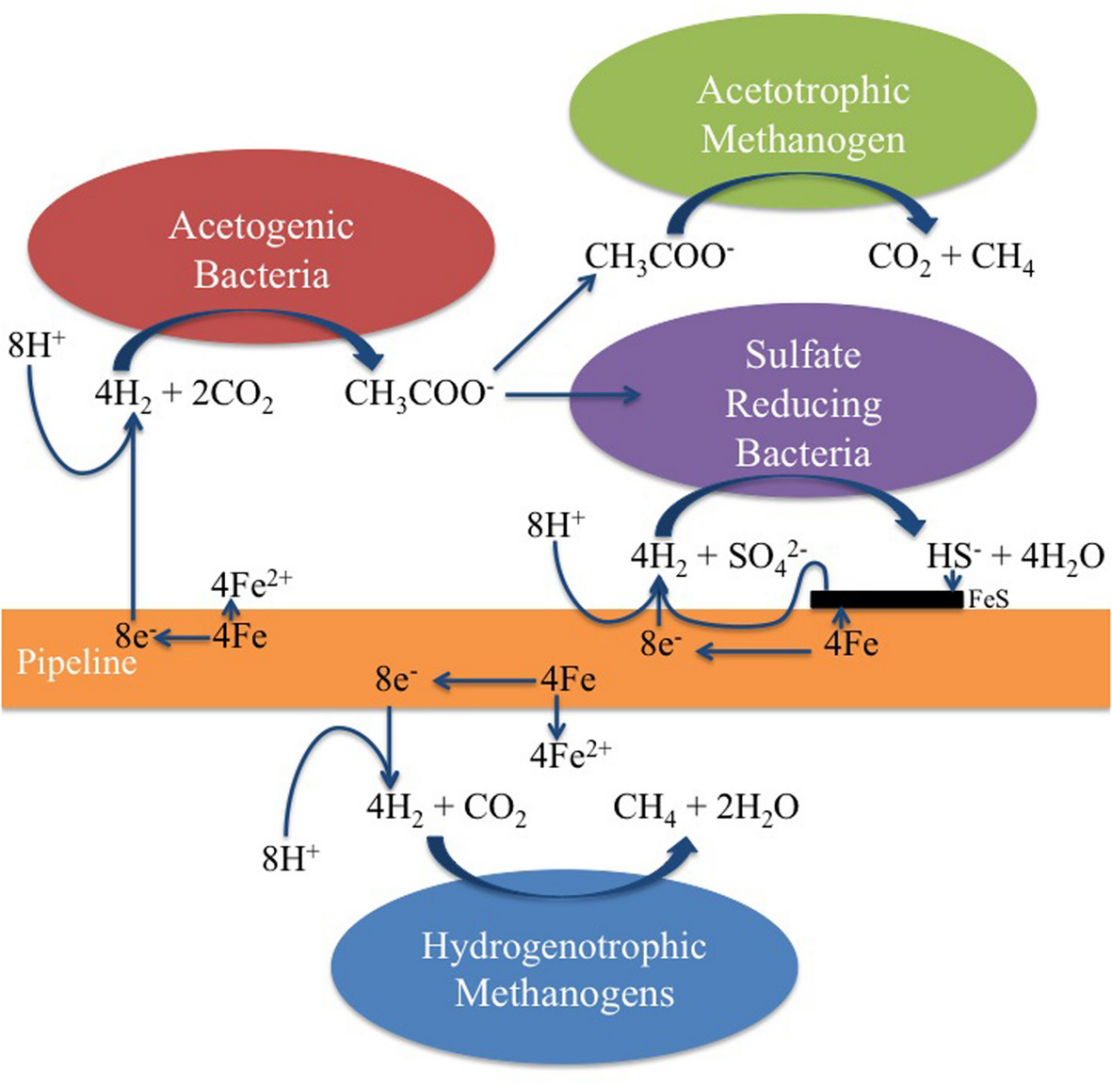
**Hydrogenotrophic microorganisms potentially contributing to MIC**. Note that the acetate formed by acetogenic bacteria can be used as a carbon source by the SRB. Although the uptake of dihydrogen (H_2_) is suggested here, direct uptake of electrons from the steel surface by SRB and methanogens has also been suggested (Dinh et al., 2004; Enning et al., 2012).

These features are captured in the extended MIC model shown in Figure 9. We have shown the uptake of reducing equivalents (H^+^ + e^−^) as dihydrogen (H_2_), realizing that direct uptake of electrons is another mechanism that has been proposed (Dinh et al., 2004; Enning et al., 2012). Accordingly, hydrogenotrophic acetogens, methanogens, or SRB could all contribute directly to corrosion by using metallic iron as an electron donor. The combined action of hydrogenotrophic acetogens and acetotrophic methanogens would be the same as that of hydrogenotrophic methanogens alone. In the coupon-attached biofilms for P0866, P0866S, and P0848S, the hydrogenotrophic *Methanobacteriaceae* predominated (Table 2: 71.75–89.31%), whereas the PW7 biofilm had a high fraction of *Acetobacterium* (2.93%) and of the acetotrophic *Methanosaeta* (17.14%).

We should point out that an acetogen pure culture, capable of deriving energy for growth from corroding iron has not yet been isolated. The suggested involvement of acetogens in corrosion thus rests on the following observations: (i) acetate production from steel upon incubation with environmental samples (Figures 2–5), (ii) the presence of acetate at the pipeline surface (Table 1: 19 ppm in PW8-PAS, higher than in the other field samples), and (iii) the high fraction of *Acetobacterium* at the pipeline surface compared to the other field samples (Table 2: 5.56% in PW8-PAS, but only 0–0.55% for the other samples). Except for the P0866 enrichment biofilm, the fraction of *Acetobacterium* was also high in the biofilm samples (Table 2: 2.93–4.86%). Acetate production by acetogens and the potential downstream effects on MIC are shown in Figure 9.

In water-transporting pipelines lacking organic carbon, the environmental role of corrosive acetogens may be to provide critical organic metabolites required. The acetate produced by acetogens through steel corrosion is used as an energy substrate by acetotrophic methanogens in present field samples. It may also be used as a carbon source by hydrogenotrophic methanogens, just as for hydrogenotrophic SRB. Incompletely-oxidizing SRB, those which oxidize lactate to acetate and CO_2_, are incapable of growth with only CO_2_ as the carbon source (Badziong et al., 1979; Rabus et al., 2006). These SRB form pyruvate from acetate and CO_2_ in the presence of ATP and H_2_ as the first step in their carbon metabolism under these conditions. The extended MIC scheme (Figure 9) indicates that the formation of acetate from CO_2_ by acetogens using the reducing power of iron allows these incompletely-oxidizing SRB to grow. Consequently a pipeline transporting water with little or no organic carbon, but containing bicarbonate and sulfate would still be subject to SRB-mediated corrosion, as acetate is generated at the pipeline surface. In the presence of H_2_ and CO_2_ the acetogen *A. woodii* can readily supply the acetate needed for the incompletely-oxidizing SRB *D. vulgaris* to grow on H_2_ and sulfate in the absence of added acetate (Figure 7). The consortium of *A. woodii* and *D. vulgaris* is somewhat more corrosive than either strain alone (Figure 8). Corrosion by *D. vulgaris* is co-metabolic, requiring the presence of some H_2_, while sulfate is present in excess (Figure 8B). The type culture *A. woodii* was found to be unable to produce acetate when incubated with iron coupons in an N_2_-CO_2_ atmosphere, under conditions used in Figure 8C. The differences may be more significant if, in addition to a highly corrosive *Desulfovibrio ferrophilus* strain IS5 (Enning et al., 2012), a highly corrosive acetogen were to be used in these studies. Future work should be directed toward the isolation of highly corrosive acetogens.

### Conflict of interest statement

The authors declare that the research was conducted in the absence of any commercial or financial relationships that could be construed as a potential conflict of interest.

## References

[B1] Badziong W. Ditter B. Thauer R. K. (1979). Acetate and carbon dioxide assimilation by *Desulfovibrio vulgaris* (Marburg), growing on hydrogen and sulfate as sole energy source. Arch. Microbiol. 123, 301–305 10.1007/BF00406665

[B2] Bihari Z. Szvetnik A. Szabó Z. Blastyák A. Zombori Z. Balás M. (2011). Functional analysis of long-chain *n*-alkane degradation by *Dietzia* spp. FEMS Microbiol. Lett. 316, 100–107 10.1111/j.1574-6968.2010.02198.x 21204932

[B3] Caffrey S. M. Park H. S. Been J. Gordon P. Sensen C. W. Voordouw G. (2008). Gene expression by the sulfate-reducing bacterium *Desulfovibrio vulgaris* Hildenborough grown on an iron electrode under cathodic protection conditions. Appl. Environ. Microbiol. 74, 2404–2413 10.1128/AEM.02469-07 18310429 PMC2293145

[B4] Caffrey S. M. Park H. S. Voordouw J. K. He Z. Zhou J. Voordouw G. (2007). Function of periplasmic hydrogenases in the sulfate-reducing bacterium *Desulfovibrio vulgaris* Hildenborough. J. Bacteriol. 189, 6159–6167 10.1128/JB.00747-07 17601789 PMC1951932

[B5] Callbeck C. M. Agrawal A. Voordouw G. (2013). Acetate production from oil under sulfate-reducing conditions in bioreactors injected with sulfate and nitrate. Appl. Environ. Microbiol. 79, 5059–5068 10.1128/AEM.01251-13 23770914 PMC3754712

[B6] Daniels L. Belay N. Rajagopal B. S. Weimer P. J. (1987). Bacterial methanogenesis and growth from CO_2_ with elemental iron as the sole source of electrons. Science 237, 509–511 10.1126/science.237.4814.509 17730323

[B7] Dinh H. T. Kuever J. Mußmann M. Hassel A. W. (2004). Iron corrosion by novel anaerobic microorganisms. Lett. Nat. 427, 829–832 10.1038/nature02321 14985759

[B8] Enning D. Venzlaff H. Garrelfs J. Dinh H. T. Meyer V. Mayrhofer K. (2012). Marine sulfate-reducing bacteria cause serious corrosion of iron under electroconductive biogenic mineral crust. Environ. Microbiol. 14, 1772–1787 10.1111/j.1462-2920.2012.02778.x 22616633 PMC3429863

[B9] Finster K. W. Kjeldsen K. U. Kube M. Reinhardt R. Mußmann M. Amann R. (2013). Complete genome sequence of *Desulfocapsa sulfexigens*, a marine deltaproteobacterium specialized in disproportionating inorganic sulfur compounds. Stand. Genomic Sci. 8, 58–68 10.4056/sigs.3777412 23961312 PMC3739170

[B10] George K. Nesic S. de Waard C. (2004). Electrochemical investigation and modeling of carbon dioxide corrosion of carbon steel in the presence of acetic acid, in NACE International, Corrosion 2004 Conference and Expo, NACE International, Paper no. 04379 (Houston, TX).

[B11] Hedges B. McVeigh L. (1999). The role of acetate in CO_2_ corrosion: the double whammy, in NACE International Corrosion 1999 Conference and Expo, NACE International, Paper no. 21 (Houston, TX).

[B12] Hu P. Rismani-Yazdi H. Stephanopoulos G. (2013). Anaerobic CO_2_ fixation by the acetogenic bacterium *Moorella thermoacetica*. AlChE J. 59, 3176–3183 10.1002/aic.14127

[B13] Leang C. Ueki T. Nevin K. P. Lovley D. R. (2013). A genetic system for *Clostridium ljungdahlii*: a chassis for autotrophic production of biocommodities and a model homoacetogen. Appl. Environ. Microbiol. 79, 1102–1109 10.1128/AEM.02891-12 23204413 PMC3568603

[B14] Liu D. Chen Z. Y. Guo X. P. (2008). The effect of acetic acid and acetate on CO_2_ corrosion of carbon steel. Anticorros. Methods Mater. 55, 130–134 10.1108/00035590810870437

[B15] Park H. S. Chatterjee I. Dong X. Wang S.-H. Sensen C. W. Caffrey S. M. (2011). Effect of sodium bisulfite injection on the microbial community composition in a brackish-water transporting pipeline. Appl. Environ. Microbiol. 77, 6908–6917 10.1128/AEM.05891-11 21856836 PMC3187121

[B16] Phelps T. J. (1991). Similarity between biotransformation rates and turnover rates of organic matter biodegradation in anaerobic environments. J. Microbiol. Methods 13, 243–254 10.1016/0167-7012(91)90061-T23763330

[B17] Rabus R. Hansen T. A. Widdel F. (2006). Dissimilatory sulfate- and sulfur-reducing prokaryotes, in The Prokaryotes, eds Dworkin M. Falkow S. Rosenberg E. Schleifer K.-H. Stackebrandt E. (New York, NY: Springer), 659–768 10.1007/0-387-30742-7_22

[B18] Schiel-Bengelsdorf B. Dürre P. (2012). Pathway engineering and synthetic biology using acetogens. FEBS Lett. 586, 2191–2198 10.1016/j.febslet.2012.04.043 22710156

[B19] Soh J. Dong X. Caffrey S. M. Voordouw G. Sensen C. W. (2013). Phoenix 2: a locally installable large-scale 16S rRNA gene sequence analysis pipeline with web interface. J. Biotechnol. 167, 393–403 10.1016/j.jbiotec.2013.07.004 23871656

[B20] Straub M. Demler M. Weuster-Botz D. Dürre P. (2014). Selective enhancement of autotrophic acetate production with genetically modified *Acetobacterium woodii*. J. Biotechnol. 178, 67–72 10.1016/j.jbiotec.2014.03.005 24637370

[B21] Suflita J. M. Phelps T. J. Little B. (2008). Carbon dioxide corrosion and acetate: a hypothesis on the influence of microorganisms. Corros. Sci. 64, 854–859 10.5006/1.3279919

[B22] Trüper H. G. Schlegel H. G. (1964). Sulphur metabolism in Thiorhodaceae I. Quantitative measurements on growing cells of *Chromatium okenii*. Antonie van Leeuwenhoek 30, 225–238 10.1007/BF02046728 14218435

[B23] Uchiyama T. Ito K. Mori K. Tsurumaru H. Harayama S. (2010). Iron-corroding methanogen isolated from a crude-oil storage tank. Appl. Environ. Microbiol. 76, 1783–1788 10.1128/AEM.00668-09 20118376 PMC2838011

